# Accuracy-Enhanced Calibration Method for Robot-Assisted Laser Scanning of Key Features on Large-Sized Components

**DOI:** 10.3390/s25247518

**Published:** 2025-12-10

**Authors:** Zhilong Zhou, Xu Zhang, Xuemei Sun, Faqiang Xia, Jinhao Zeng

**Affiliations:** College of Mechanical and Vehicle Engineering, Linyi University, Linyi 276000, China; 17753854071@163.com (X.Z.); sunxuemei@lyu.edu.cn (X.S.); m15664567816@163.com (F.X.); xiu_cheng123@163.com (J.Z.)

**Keywords:** accuracy-enhanced calibration, robot-assisted laser scanning hybrid measurement, extrinsic parameter calibration, large-scale spatial measurement field, geometric constraints, error control

## Abstract

In advanced manufacturing, accurate and reliable 3D geometry measurement is vital for the quality control of large-sized components with multiple small key local features. To obtain both the geometric form and spatial position of these local features, a hybrid robot-assisted laser scanning strategy is introduced, combining a laser tracker, a fringe-projection 3D scanner, and a mobile robotic unit that integrates an industrial robot with an Automated Guided Vehicle. As for improving the overall measurement accuracy, we propose an accuracy-enhanced calibration method that incorporates both error control and compensation strategies. Firstly, an accurate extrinsic parameter calibration method is proposed, which integrates robust target sphere center estimation with distance-constrained-based optimization of local common point coordinates. Subsequently, to construct a high-accuracy, large-scale spatial measurement field, an improved global calibration method is proposed, incorporating coordinate optimization and a hierarchical strategy for error control. Finally, a robot-assisted laser scanning hybrid measurement system is developed, followed by calibration and validation experiments to verify its performance. Experiments verify its high precision over 14 m (maximum error: 0.117 mm; mean: 0.112 mm) and its strong applicability in large-scale scanning of key geometric features, providing reliable data for quality manufacturing of large-scale components.

## 1. Introduction

The manufacturing capabilities of high-end equipment used in major projects such as aviation, aerospace, maritime engineering, and energy serve as a critical indicator of a nation’s technological advancement [1,2]. Large-sized components (LSCs) serve as the core load-bearing structure of high-end equipment, featuring large size and complex structure. Among them, the small-scale key local features (KLFs, 1 × 1 mm to 25 × 25 mm) on LSCs, such as feature holes and feature surfaces, are distributed discretely over a wide area (2 to 12 m). Ensuring precise and automated 3D shape measurement of these features plays a vital role in quality assurance, product reliability improvement, and cost reduction in manufacturing processes [3,4]. For instance, in the production process of large-sized components, in order to ensure high-precision integrated manufacturing, it is necessary to conduct online detection of key features and use the detection results to guide the subsequent manufacturing process. Moreover, to ensure the assembly accuracy, cross-scale high-precision inspection of key features is essential to avoid forced assembly caused by geometric interference at connection interfaces. Therefore, the research on high-precision online detection methods for local small-scale key features within a large-scale space is of great significance for ensuring the high-quality and reliable manufacturing of large-sized components.

With the increasing extremity in both the size and manufacturing precision of core structural components, the measurement requirements for their key features, such as size and accuracy, have also shown a polarized trend. Consequently, hybrid measurement methods that combine large-scale spatial coordinate measurement systems with small-range vision-based systems have become an essential means. According to the measurement principle, the main large-sized spatial coordinate measurement systems include laser tracker [5], total station [6], indoor global position system (i-GPS) [7], laser radar [8], photogrammetry [9], etc. Among small-range vision-based measurement systems, those based on structured light are the most widely used. To guarantee reliable measurement of key local features (KLFs), the hybrid measurement system (HMS) undergoes a series of calibration steps prior to data acquisition: (1) camera calibration, (2) intrinsic calibration of the 3D scanner, (3) local extrinsic parameter calibration, and (4) global calibration for constructing the measurement field. Within the HMS, extrinsic parameter calibration establishes the spatial transformation between the 3D scanner’s coordinate system and an intermediate reference frame. Within the global measurement domain, global calibration plays a critical role in minimizing cumulative errors in final measurements and maintaining overall accuracy across large-scale workspaces. Because camera and 3D scanner calibration is usually predefined, system setup primarily focuses on performing global and local calibration.

For extrinsic parameter calibration, among global direct pose estimation methods in structured-light vision measurement systems, the extrinsic parameter calibration method based on cooperative targets is the most widely used [10]. Barone et al. [11,12] utilized a two-dimensional planar checkerboard calibration plate, combined with Singular Value Decomposition (SVD), to perform the extrinsic parameter calibration of a hybrid measurement system integrating a 3D structured-light scanner with global stereo vision. Liu et al. [13] combined the indirect calibration method based on rigid body transformation to achieve the rapid calibration of extrinsic parameters. Du et al. [14] utilized target balls that could be simultaneously identified by both global and local measurement devices as cooperative targets. Based on the single-point multiple-point method and the rotation method, they constructed common feature points with non-coplanar distribution and pose reference coordinate systems, respectively, achieving the rapid calculation of the extrinsic parameters matrix of the 3D scanner. To enhance the calibration accuracy of extrinsic parameters, Qu et al. [15] designed a cooperative target and combined the adjustment optimization technology to establish a calibration model that joins multiple independent reference frame transformations, achieving high-accuracy and rapid calibration of extrinsic parameters. However, it did not take into account the issue of differences in measurement data accuracy levels. To address this issue, Jiang et al. [16,17,18] proposed an extrinsic parameter calibration method that takes into account the scale factor. This method first established a coordinate fusion model for the combined measurement of global binocular vision and local optical scanning, and then, based on numerical simulation, explored the influence law of scale differences on the global positioning accuracy, established an extrinsic parameter calibration model considering the scale factor, and combined multi-view adjustment optimization technology to enhance the calibration accuracy of extrinsic parameters. Garcia-D’Urso et al. [19] proposed a method for estimating multi-camera extrinsic parameters based on structured 3D markers and iterative optimization. By integrating AI-driven deep learning or regression modules, it offers significant advantages in feature extraction, outlier removal, and initial value estimation, thereby enhancing the robustness and automation level of external parameter solution. Pan et al. [20] proposed an online extrinsic calibration method that unifies LiDAR and camera data into depth-map representations and leverages depth-edge discontinuities as robust geometric constraints. By incorporating a miscalibration detection module and on-manifold optimization for continuous parameter refinement, the method effectively addresses vibration- and deformation-induced extrinsic drift and enhances the resilience and autonomy of multi-sensor calibration in dynamic environments. This line of work highlights emerging AI- and optimization-driven approaches for achieving resilient, autonomous extrinsic calibration in dynamic environments. In summary, current studies on extrinsic parameter calibration of terminal measurement systems have achieved significant results in aspects such as the construction of calibration targets and algorithms for solving extrinsic parameters. However, the critical challenges of severe accumulation of multi-baseline transformation errors caused by the difficulty in tracing, constraining, and compensating heterogeneous measurement errors remain effectively unresolved. Moreover, multi-reference frame transformations involve large-angle rotations, which lead to poor stability in the calculation of transformation parameters, making it difficult to ensure solution accuracy.

For global calibration, constructing a large-scale global measurement field based on laser tracker is one of the most effective means for achieving global accuracy control. Among its key challenges, the measurement error control of global common points (GCPs) is the key research direction to ensure the accuracy of such field construction [21]. Jin et al. [22] elaborated in detail on the construction method of a large-scale measurement field using station-transfer method based on a laser tracker and established a transfer station error model that takes into account both the global common point configuration and measurement errors. The study revealed the correlation between the layout of common points, the measurement errors of the laser tracker, the parameter errors of the transfer station, and the transfer station errors. Predmore [23] proposed a method for optimizing the coordinate transformation parameters considering the ellipsoid of the measurement uncertainty of the GCP, which utilized the Marhalobanb beam adjustment technique to solve the orientation problem of the multi-base station measurement system and the weighted optimization of the common point coordinate measurements. To address the issue of unknown and uncontrollable measurement errors in GCPs, Wang et al. [24] proposed a large-scale coordinate unification method based on standard artifact. This method first introduces standard artifact with prior geometric knowledge to replace the traditional independent common points and eliminates gross errors by setting allowable error limits, thereby obtaining qualified initial values for common reference point measurements. On this basis, a coordinate value optimization method based on geometric constraints is proposed, further improving the measurement accuracy of common reference points. Additionally, through the Procrustes spatial coordinate system registration method, the coordinate unification of large-scale measurement systems was achieved. However, this method cannot effectively reduce the impact of laser tracker angle measurement errors on the measurement accuracy of spatial points. To improve the global measurement accuracy in the combined measurement of large-sized and complex components, Lin et al. [25] developed a strategy to establish a large-scale spatial measurement field based on laser interferometric distance constraints. The approach initially applied a multi-station measurement scheme to derive the preliminary 3D coordinates of the GCPs, followed by singular value decomposition (SVD) to determine the stations’ initial orientation. An error model incorporating high-precision laser interferometric distance constraints is established to mitigate the impact of angular measurement errors from the laser tracker on spatial point accuracy. In addition, by introducing multiple one-dimensional carbon fiber rods to form spatial length references, a precision enhancement strategy based on spatial distance constraints is proposed. Length and angular constraint equations are formulated, and a nonlinear optimization algorithm is applied to calculate the correction values of the reference point coordinates, thereby refining the transfer errors and improving measurement accuracy in localized areas. Fan et al. [26], based on the construction of the weighted rank deficiency adjustment model for laser interferometry, introduced a known-length reference ruler, constructed length constraints for a single posture, and established an adjustment model with additional constraints, thereby further improving the overall accuracy of point distribution across the measurement field. However, under complex on-site conditions, the aforementioned methods face significant challenges in simultaneously identifying and controlling outlier measurements, which limits their ability to effectively suppress the influence of angular measurement errors from laser trackers on spatial point accuracy. Recent advances in large-scale metrology have also highlighted the importance of dynamic error compensation and multi-station self-calibration for laser tracker networks. Zou et al. [27] proposed a high-precision construction strategy for multi-station laser tracker measurement networks, incorporating ERS-based weighted optimization, iterative refinement of inter-station transformation parameters, and optimal LTMS configuration planning (including station number and spatial placement) to minimize global network error. This line of research demonstrates the growing emphasis on intelligent, optimization-driven frameworks for improving large-scale automated measurement accuracy. Ma et al. [28] introduced an enhanced registration strategy that integrates Enhanced Reference System (ERS) point-weighted self-calibration with thermal deformation compensation, significantly improving the stability of multi-station global measurements under varying environmental conditions. However, due to the limitations of the difficulty in constructing multi-domain and multi-pose geometric constraints and the insufficient constraints of external high-precision features, it is difficult to effectively control the global datum accuracy under the restricted layout.

To address the aforementioned limitations, we introduce an accuracy-enhanced calibration method that integrates error control and compensation strategies. Firstly, an accurate extrinsic parameter calibration method is proposed, which integrates robust target sphere center estimation with distance-constrained-based optimization of local common point coordinates. This method establishes a correction model for reference frame transformation parameters, effectively compensating for multi-source heterogeneous measurement errors. Subsequently, to construct a high-accuracy large-scale spatial measurement field, an improved global calibration method is proposed. This method incorporates coordinate measurement value optimization and applies a hierarchical strategy for measurement error control. Finally, a robot-assisted laser scanning hybrid measurement system was developed, followed by calibration and validation experiments to verify its performance.

This paper is organized as follows. Section 2 presents the proposed measurement method along with its fundamental principles. Section 3 describes the procedure for calibrating the extrinsic parameters of the 3D scanner. Section 4 illustrates the method for constructing a large-scale spatial measurement field. Section 5 presents the calibration and validation experiments, whereas Section 6 summarizes the key outcomes and future work of the study.

## 2. Measurement Method and Principle

### 2.1. Overview of Robot-Assisted Laser Scanning Hybrid Measurement System

**Figure 1:**
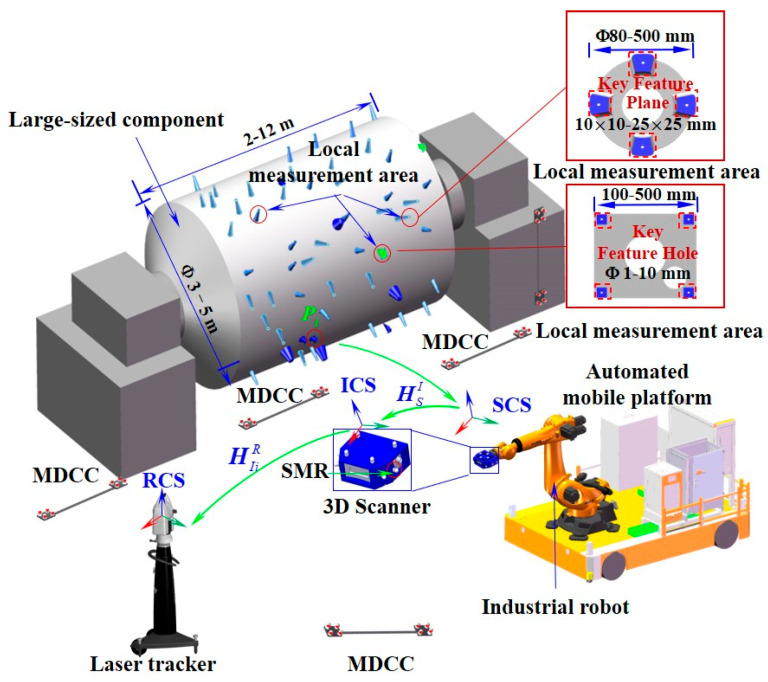
Schematic overview of robot-assisted laser scanning hybrid measurement system.

The proposed robot-assisted laser scanning hybrid measurement system (RLSHS) is composed of three essential modules: a laser tracker responsible for global reference acquisition, a structured-light 3D scanner for local geometry capture, and a mobility-enabled robotic platform that integrates an industrial robot with an automated mobile platform. A schematic overview is presented in Figure 1.

Automated and high-accuracy 3D measurement of KLFs plays a pivotal role in ensuring manufacturing quality while reducing inspection time and manual effort. Given the extensive measurement range of LSCs, the multiscale nature of their features, and the need for high accuracy in both shape and position, as well as multi-source data integration, a laser tracker is employed as the global coordinate measurement device. This enables long-range measurements while maintaining consistency between global and local accuracy. In addition, a fringe-projection-based 3D scanner is employed for local terminal measurements, ensuring high accuracy in close-range inspections. The measurement system integrates a mobile robotic unit consisting of an industrial manipulator and a mobile platform, thereby enhancing both operational efficiency and flexibility. System coordination is achieved through industrial Fieldbus networks and dedicated communication software, forming a robust auxiliary control framework. The scanning terminal, integrating a 3D scanner and several spherically mounted retroreflectors (SMRs), is installed on the end-effector of an industrial robot, which serves as the main measurement actuator by combining motion flexibility with high positioning repeatability. This configuration allows accurate positioning of the 3D scanner to acquire KLF information in segmented work areas. Moreover, a mobile platform transports the actuator across larger workspaces, thereby enlarging the effective measurement range of the proposed RLSHS.

In the proposed RLSHS, the laser tracker, mobile platform, and 3D scanner operate in a real-time cooperative manner. The measurement process of the system is as follows: Firstly, before the formal measurement, a large-scale spatial measurement field (spatial measurement network) is constructed by combining laser tracker transfer station technology with several multi-dimensional cooperative calibrators (MDCC), and a high-precision global measurement reference is established. The global measurement network is established only once prior to system operation, functioning as a stable, reusable geometric reference throughout all subsequent measurements; thus, its setup time is not part of the per-task efficiency metric. At the initial stage of formal measurement, the mobile platform first transports the robot to the vicinity of the target region, after which the laser tracker continuously measures the SMRs mounted on the 3D scanner and robot to provide global pose feedback. Using this real-time guidance, the robot performs fine positioning of the scanner along predefined viewpoints. Before each scan, the laser tracker verifies pose stability, ensuring that the scanner captures the target area under a globally referenced coordinate frame. Through this closed-loop interaction, the system maintains coherent coordination of global referencing, robot motion, and high-accuracy 3D data acquisition.

### 2.2. Measurement Model

The coordinate systems of the proposed RLSHS primarily consist of three frames: the laser tracker coordinate system $O_{R}-X_{R}Y_{R}Z_{R}$ (RCS), the coordinate system of the dynamic 3D scanner $O_{s}-X_{s}Y_{s}Z_{s}$ (SCS), and the intermediate coordinate system $O_{I}-X_{I}Y_{I}Z_{I}$ (ICS), which defines the reference pose of the local 3D scanner. As illustrated in Figure 1, three transformation matrices are involved among these coordinate systems: matrix $H_{S}^{I}$ describes the transformation from ICS to SCS, matrix $H_{I}^{R}$ from RCS to ICS, and matrix $H_{S}^{R}$ from RCS to SCS. Specifically, the ICS serves as an intermediate frame bridging the RCS and the SCS. To establish this reference frame, multiple sophisticated magnetic nests (SMNs) for fixing SMRs are rigidly mounted on the scanner fixture. The relative positions of these reference observation points are pre-calibrated using a high-precision coordinate measuring machine (CMM). During operation, the laser tracker measures the positions of the SMRs to determine the pose of the scanner and to enable accurate real-time tracking.

Let $P_{i}$ denote a point located on the key geometric feature of a large-sized component, and $P_{i}^{S}=\left(x_{i}^{S},y_{i}^{S},\;z_{i}^{S}\right)$ represent its corresponding 3D coordinates in the SCS. These can be transformed into the ICS through the following formula:

(1) $$P_{i}^{I}=R_{S}^{I}P_{i}^{S}+T_{S}^{I}$$

where $R_{S}^{I}$ and $T_{S}^{I}$ represent the rotation matrix and translation vector of matrix $H_{S}^{I}$, respectively. Matrix $H_{S}^{I}$, also referred to as the extrinsic parameter matrix of the 3D scanner, is a fixed transformation matrix.

Usually, any station RCS can be designated as the global coordinate system (GCS), and the point coordinates defined in the ICS are transformed into the GCS using the following transformation:

(2) $$P_{i}^{G}=R_{I}^{G}P_{i}^{I}+T_{I}^{G}$$

By combining Equations (1) and (2), a cross-scale measurement data transfer model is established as follows:

(3) $$P_{i}^{G}=\left(R_{I}^{G}R_{S}^{I}\right)P_{i}^{S}+\left(R_{I}^{G}T_{S}^{I}+T_{I}^{G}\right)$$

where $R_{S}^{G}=R_{I}^{G}R_{S}^{I}$ represents the rotation matrix of the SCS with respect to the GCS and $T_{I}^{G}=R_{I}^{G}T_{S}^{I}+T_{I}^{G}$ denotes the corresponding translation vector.

The measurement of KLFs in LSCs presents several challenges, including uncertain measurement deviations and uncontrollable calibration errors. To enhance the overall measurement accuracy, a series of calibration procedures was conducted prior to deploying the RLSHS for KLF inspection. First, the matrix $H_{S}^{I}$ was obtained through the extrinsic parameters’ calibration. Second, global calibration was performed to minimize alignment errors among local datasets and to establish a unified large-scale spatial measurement field. Specifically, the extrinsic parameter calibration was categorized as local calibration (refer to Section 3), while the construction of the large-scale spatial measurement field was considered global calibration (see Section 4).

## 3. Extrinsic Parameter Calibration

A high-precision coordinate transformation model $H_{S}^{I}$ between the SCS and ICS is constructed through extrinsic parameter calibration. As calibration is required before the RLSHS is applied to large-scale measurement tasks and cannot be repeated once operation begins, the reliability of the entire measurement process critically depends on the calibration accuracy. To suppress transformation errors, we propose a refined extrinsic calibration approach that combines robust estimation of target sphere centers with a distance-constrained optimization of local common point (LCP) coordinates.

### 3.1. Extrinsic Parameter Calibration Model

**Figure 2:**
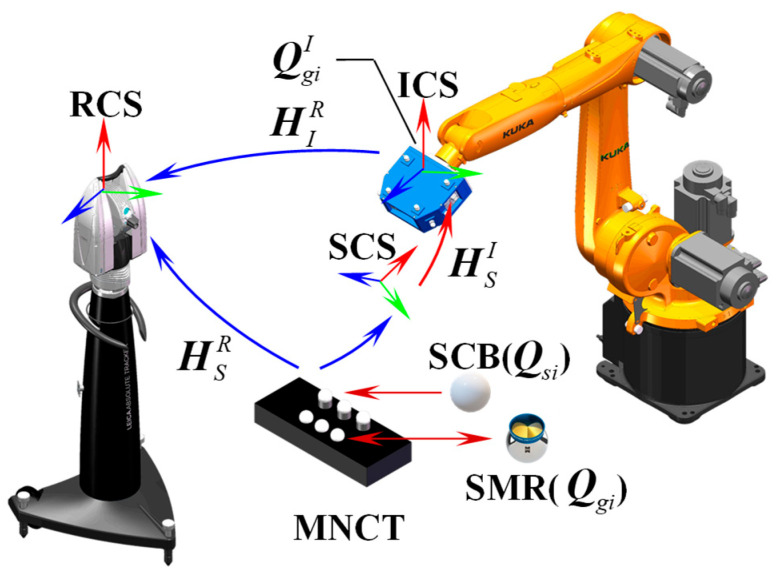
Schematic of calibration principle based on MNCT.

During the calibration process, a specially designed multi-source, non-coplanar cooperative target (MNCT), comprising several SMRs and standard ceramic balls (SCBs), was fixed within the measurement workspace. A schematic of the calibration procedure is shown in Figure 2.

Let $P_{si}^{S}=\left(x_{si}^{S},\;y_{si}^{S},\;z_{si}^{S}\right)$ denote the 3D coordinates of the observed target point center $Q_{si},\;si=s1,\;s2,\;\cdots ,\;sn$ on the MNCT in the SCS, and let $P_{gi}^{R}=\left(x_{gi}^{R},\;y_{gi}^{R},\;z_{gi}^{R}\right)$ denote the corresponding coordinates of the same target point center $Q_{gi},\;gi=g1,\;g2,\;\cdots ,\;gn$ in the RCS. Based on the principle of coordinate transformation, the following equation is established:

(4) $$\left[\begin{array}{c} P_{gi}^{R}\\ 1 \end{array}\right]=H_{S}^{R}\left[\begin{array}{c} P_{si}^{S}\\ 1 \end{array}\right]=\left[\begin{array}{cc} R_{S}^{R} & T_{S}^{R}\\ 0 & 1 \end{array}\right]\left[\begin{array}{c} P_{si}^{S}\\ 1 \end{array}\right]$$

Let $P_{i}^{I}=\left(x_{i}^{I},\;y_{i}^{I},\;z_{i}^{I}\right)$ represent the 3D coordinates of the pose observation points $Q_{gi}^{I}$ arranged on the surface of the scanner in the ICS, and $P_{Ii}^{R}=\left(x_{Ii}^{R},\;y_{Ii}^{R},\;z_{Ii}^{R}\right)$ be the measured coordinate values of the corresponding observation points in the RCS, then the following equation can be established:

(5) $$\left[\begin{array}{c} P_{Ii}^{R}\\ 1 \end{array}\right]=H_{I}^{R}\left[\begin{array}{c} P_{i}^{I}\\ 1 \end{array}\right]=\left[\begin{array}{cc} R_{I}^{R} & T_{I}^{R}\\ 0 & 1 \end{array}\right]\left[\begin{array}{c} P_{i}^{I}\\ 1 \end{array}\right]$$

By combining Equations (3)–(5), the transformation matrix between SCS and ICS is obtained:

(6) $$H_{S}^{I}={\left(H_{I}^{R}\right)}^{-1}H_{S}^{R}=\left[\begin{array}{cc} {\left(R_{I}^{R}\right)}^{-1}R_{S}^{R} & {\left(R_{I}^{R}\right)}^{-1}\left(T_{S}^{R}-T_{S}^{R}\right)\\ 0 & 1 \end{array}\right]$$

The coordinate values of the target points obtained by the measurement equipment contain measurement errors, which lead to deviations in each stage of reference frame transformation. To improve the estimation accuracy of transformation parameter, it is necessary to reduce the multi-source heterogeneous measurement errors, thereby establishing an optimization calibration model that jointly constrains the errors of each independent transformation, that is:

(7) $${\tilde{H}}_{S}^{I}={\left({\tilde{H}}_{I}^{R}\right)}^{-1}{\tilde{H}}_{S}^{R}$$

where ${\tilde{H}}_{S}^{I}$, ${\tilde{H}}_{I}^{R}$ and ${\tilde{H}}_{S}^{R}$ represent the optimized values of $H_{S}^{I}$, $H_{I}^{R}$ and $H_{S}^{R}$, respectively.

### 3.2. Robust Target Sphere Center Estimation via Denoised Point Cloud Fitting

**Figure 3:**
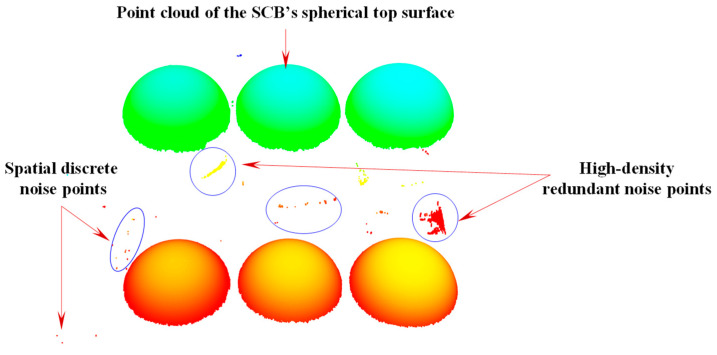
Original point cloud of SCB acquired by the 3D scanner.

As mentioned earlier, the three-dimensional coordinate values of the center of the scanning target ball (SCB) in the SCS are one of the important input coordinate values for solving the transformation matrix $H_{S}^{R}$, and the measurement accuracy directly affects the accuracy of solving the transformation parameters. During the measurement process, a 3D scanner was used to scan the SCB, and the obtained original point cloud of the SCB’s spherical top surface is shown in Figure 3.

Due to the large volume of point cloud data and the presence of complex background noise, the 3D spatial coordinates of the SCB’s center cannot be directly obtained. Therefore, a point cloud preprocessing algorithm is required to process the SCB’s scanned data and accurately fit the center coordinates of the sphere. However, in the scanned data, there are various types of complex noises, such as high-density redundant points and sparse spatial discrete points, which affect the fitting accuracy of the sphere’s center. To address this issue, this study investigates a scanning target ball center-fitting method based on point cloud denoising. This method uses the radius filtering method, the random sample consensus (RANSAC) algorithm, and the Euclidean clustering segmentation method to remove noise and accurately fit the coordinates of the ball center, providing a reliable data basis for the subsequent solution of the reference transformation matrix.

**Figure 4:**
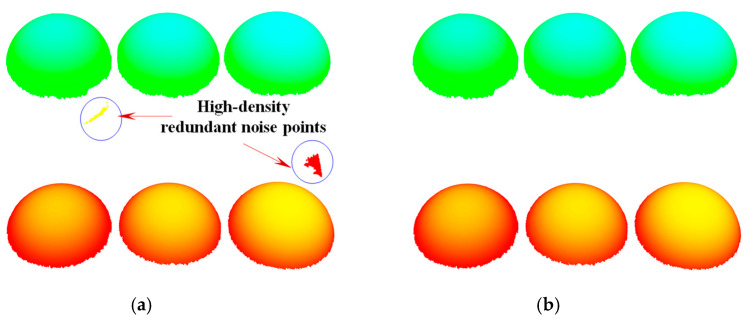
Point cloud after noise reduction. (**a**) point cloud obtained after removing sparse space discrete points, and (**b**) point cloud obtained after removing high-density redundant noise points.

First, to improve processing efficiency of the point cloud and ensure the accuracy of sphere center fitting, the initial point cloud is subjected to downsampling. Based on this, radius filtering method is applied to eliminate the sparse space discrete points, obtaining the sphere top surface point cloud that only contains high-density redundant noise points, as shown in Figure 4a. Further, for the high-density redundant noise points, considering that their point cloud density is basically the same as that of the sphere’s top surface point cloud, the Random Sample Consensus (RANSAC) algorithm is utilized to fit them into a plane and eliminate them, yielding the point cloud of the sphere’s top surface, as shown in Figure 4b.

**Figure 5:**
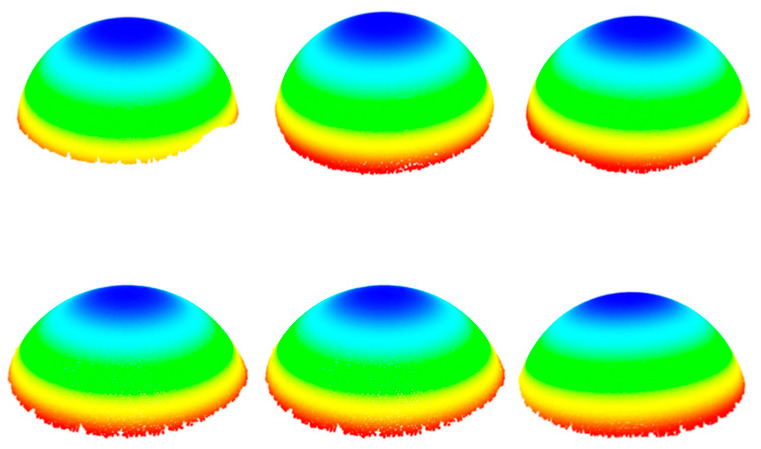
Point cloud of each SCB after point cloud segmentation.

Subsequently, the point cloud is segmented into multiple groups of individual target spherical point clouds using the Euclidean clustering segmentation method, as shown in Figure 5. Finally, the RANSAC algorithm is applied to fit the spherical surfaces, yielding the coordinates of the SCB sphere centers.

### 3.3. Distance-Constraint-Based Optimization Method for Local Common Point Coordinates

To address the difficulties in tracing, constraining and compensating for the multi-source heterogeneous measurement errors during the extrinsic parameter calibration, a coordinate optimization method based on multi-distance constraints is proposed. This method utilizes the prior distances between target points on the MNCT as reference values. First, a constraint model is formulated using these known distances. Then, the model is linearized, and the spatial relationships between measurement points are represented as a system of equations. Finally, conditional least-squares adjustment is applied to estimate coordinate corrections, thereby compensating for errors from heterogeneous sources.

**Figure 6:**
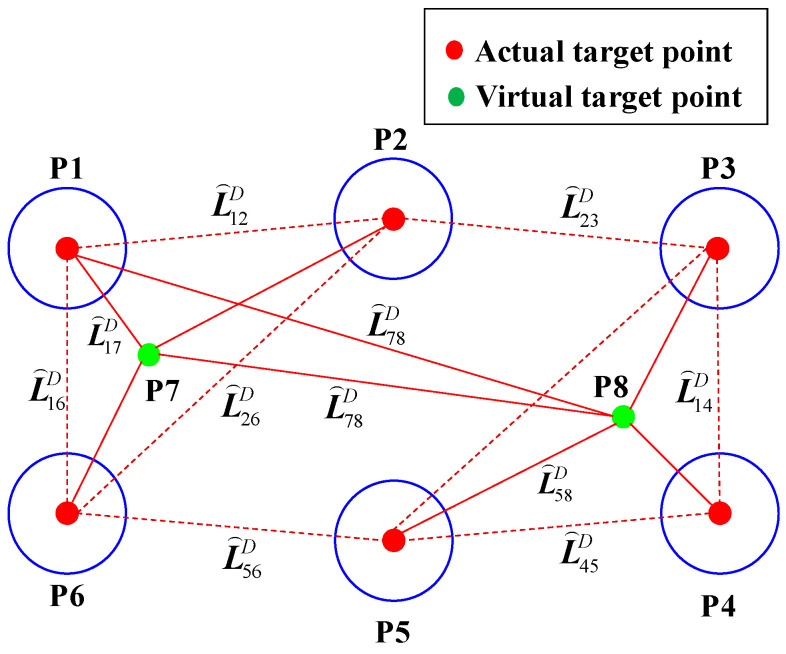
Schematic representation of constraint distances defined by actual and virtual points.

According to the principles of conditional adjustment, when the number of constraint distance equations must be greater than the number of coordinates to be solved, that is, the number of local common points is no less than 7, there will be redundant equations. To reduce the number of actual physical targets, this study introduces a hybrid layout strategy that incorporates both real and virtual points. This method determines a virtual point coordinate value by calculating the coordinates of three actual points and then constructs constraint distances, as shown in Figure 6. Finally, it realizes the optimization of coordinate measurement value errors.

This section takes the local common points on the MNCT as an example to elaborate on the coordinate value optimization method proposed in this study. Denote $X^{M}=\left[x_{1}^{M},\;y_{1}^{M},\;\cdots ,\;y_{n}^{M},\;z_{n}^{M}\right]$ as the measured three-dimensional coordinates and ${\overset{̑}{X}}^{r}=\left[{\overset{̑}{x}}_{1}^{r},\;{\overset{̑}{y}}_{1}^{r},\;\cdots ,\;{\overset{̑}{y}}_{n}^{r},\;{\overset{̑}{z}}_{n}^{r}\right]$ as the corresponding theoretical coordinates of the LCPs on the MNCT, acquired using either the 3D scanner or the laser tracker. Accordingly, the theoretical distance ${\overset{̑}{L}}_{ij}^{D}$ between any two LCPs can be expressed as a function of their theoretical coordinates:

(8) $$\left\{\begin{array}{c} {\left({\overset{̑}{x}}_{1}^{r}-{\overset{̑}{x}}_{2}^{r}\right)}^{2}+{\left({\overset{̑}{y}}_{1}^{r}-{\overset{̑}{y}}_{2}^{r}\right)}^{2}+{\left({\overset{̑}{z}}_{1}^{r}-{\overset{̑}{z}}_{2}^{r}\right)}^{2}-{\left({\overset{̑}{L}}_{12}^{D}\right)}^{2}=0\\ {\left({\overset{̑}{x}}_{1}^{r}-{\overset{̑}{x}}_{3}^{r}\right)}^{2}+{\left({\overset{̑}{y}}_{1}^{r}-{\overset{̑}{y}}_{3}^{r}\right)}^{2}+{\left({\overset{̑}{z}}_{1}^{r}-{\overset{̑}{z}}_{3}^{r}\right)}^{2}-{\left({\overset{̑}{L}}_{13}^{D}\right)}^{2}=0\\ \vdots \\ {\left({\overset{̑}{x}}_{i}^{r}-{\overset{̑}{x}}_{j}^{r}\right)}^{2}+{\left({\overset{̑}{y}}_{i}^{r}-{\overset{̑}{y}}_{j}^{r}\right)}^{2}+{\left({\overset{̑}{z}}_{i}^{r}-{\overset{̑}{z}}_{j}^{r}\right)}^{2}-{\left({\overset{̑}{L}}_{ij}^{D}\right)}^{2}=0\\ \vdots \\ {\left({\overset{̑}{x}}_{n-1}^{r}-{\overset{̑}{x}}_{n}^{r}\right)}^{2}+{\left({\overset{̑}{y}}_{n-1}^{r}-{\overset{̑}{y}}_{n}^{r}\right)}^{2}+{\left({\overset{̑}{z}}_{n-1}^{r}-{\overset{̑}{z}}_{n}^{r}\right)}^{2}-{\left({\overset{̑}{L}}_{n\left(n-1\right)}^{D}\right)}^{2}=0 \end{array}\right.$$

Suppose the following condition holds:

(9) $$f_{D}=\sqrt{{\left({\overset{̑}{x}}_{i}^{r}-{\overset{̑}{x}}_{j}^{r}\right)}^{2}+{\left({\overset{̑}{y}}_{i}^{r}-{\overset{̑}{y}}_{j}^{r}\right)}^{2}+{\left({\overset{̑}{z}}_{i}^{r}-{\overset{̑}{z}}_{j}^{r}\right)}^{2}}\left(i,\;j=1,\;2,\;\cdots ,\;n;\;i\neq j\right)$$

The distance constraint is linearized by performing a second-order Taylor expansion and disregarding higher-order terms:

(10) $$f_{D}=L_{ij}^{M}+\frac{\partial f_{D}}{\partial {\overset{̑}{x}}_{i}^{r}}\delta {\overset{̑}{x}}_{i}^{M}+\frac{\partial f_{D}}{\partial {\overset{̑}{y}}_{i}^{r}}\delta {\overset{̑}{y}}_{i}^{M}+\frac{\partial f_{D}}{\partial {\overset{̑}{z}}_{i}^{r}}\delta {\overset{̑}{z}}_{i}^{M}+\frac{\partial f_{D}}{\partial {\overset{̑}{x}}_{j}^{r}}\delta {\overset{̑}{x}}_{j}^{M}+\frac{\partial f_{D}}{\partial {\overset{̑}{y}}_{j}^{r}}\delta {\overset{̑}{y}}_{j}^{M}+\frac{\partial f_{D}}{\partial {\overset{̑}{z}}_{j}^{r}}\delta {\overset{̑}{z}}_{j}^{M}$$

where $L_{ij}^{M}=\sqrt{{\left(x_{i}^{M}-x_{j}^{M}\right)}^{2}+{\left(y_{i}^{M}-y_{j}^{M}\right)}^{2}+{\left(z_{i}^{M}-z_{j}^{M}\right)}^{2}}$ denotes the measured distance between two LCPs, while $\delta {\overset{̑}{X}}^{M}={\left[\delta {\overset{̑}{x}}_{i}^{M},\;\delta {\overset{̑}{y}}_{i}^{M},\;\delta {\overset{̑}{z}}_{i}^{M},\;\delta {\overset{̑}{x}}_{j}^{M},\;\delta {\overset{̑}{y}}_{j}^{M},\;\delta {\overset{̑}{z}}_{j}^{M}\right]}^{T}$ represents the correction vector accounting for their on-site measurement errors; $\left(\frac{\partial f_{D}}{\partial {\overset{̑}{x}}_{i}^{r}},\;\frac{\partial f_{D}}{\partial {\overset{̑}{y}}_{i}^{r}},\;\frac{\partial f_{D}}{\partial {\overset{̑}{z}}_{i}^{r}},\;\frac{\partial f_{D}}{\partial {\overset{̑}{x}}_{j}^{r}},\;\frac{\partial f_{D}}{\partial {\overset{̑}{y}}_{j}^{r}},\;\frac{\partial f_{D}}{\partial {\overset{̑}{z}}_{j}^{r}}\right)$ is the partial derivative.

Further, we obtain:

(11) $$\frac{\Delta x_{ij}^{0}}{L_{ij}^{M}}\delta {\overset{̑}{x}}_{i}^{M}+\frac{\Delta y_{ij}^{0}}{L_{ij}^{M}}\delta {\overset{̑}{y}}_{i}^{M}+\frac{\Delta z_{ij}^{0}}{L_{ij}^{M}}\delta {\overset{̑}{z}}_{i}^{M}-\frac{\Delta x_{ij}^{0}}{L_{ij}^{M}}\delta {\overset{̑}{x}}_{j}^{M}-\frac{\Delta y_{ij}^{0}}{L_{ij}^{M}}\delta {\overset{̑}{y}}_{j}^{M}-\frac{\Delta z_{ij}^{0}}{L_{ij}^{M}}\delta {\overset{̑}{z}}_{j}^{M}+l_{ij}^{D}=0$$

where $l_{ij}^{D}=-\left(L_{ij}^{M}-{\overset{̑}{L}}_{ij}^{D}\right)$ is the closure error.

Accordingly, Equation (11) can be expressed in matrix form as:

(12) $$A^{D}\delta {\overset{̑}{X}}^{M}-\delta L^{D}=0$$

where $A^{D}$ is the coefficient matrix; $\delta L^{D}={\left[l_{12}^{D},\;l_{13}^{D},\;\cdots ,\;l_{\left(n-1\right)n}^{D}\right]}^{T}$ is the closure error vector.

In Equation (12), there are 3*n* unknowns and $\frac{n\left(n-1\right)}{2}$ equations. By employing Lagrange multipliers to solve conditional extrema, the objective function is derived as follows:

(13) $$\Phi^{D}={\left(\delta {\overset{̑}{X}}^{M}\right)}^{T}P^{D}\delta {\overset{̑}{X}}^{M}-2{\left(K^{D}\right)}^{T}\left(A^{D}\delta {\overset{̑}{X}}^{M}-\delta L^{D}\right)$$

where $K^{D}={\left[k_{1}^{D},\;k_{2}^{D},\;\cdots ,\;k_{n}^{D}\right]}^{T}$ is the contact number vector.

Setting the first derivative of $\delta {\overset{̑}{X}}^{M}$ to zero yields:

(14) $$\frac{d\Phi^{D}}{d\left(\delta {\overset{̑}{X}}^{M}\right)}={\left(\delta {\overset{̑}{X}}^{M}\right)}^{T}P^{D}-{\left(K^{D}\right)}^{T}A^{D}=0$$

Then, by transposing both sides, the following expression is obtained:

(15) $$P^{D}\delta {\overset{̑}{X}}^{M}={\left(A^{D}\right)}^{T}{\left(K^{D}\right)}^{T}$$

Therefore, in conclusion, we can obtain:

(16) $$\left\{\begin{array}{l} K^{D}={\left(A^{D}{\left(P^{D}\right)}^{-1}{\left(A^{D}\right)}^{T}\right)}^{-1}\delta L^{D}\\ \delta {\overset{̑}{X}}^{M}={\left(P^{D}\right)}^{-1}{\left(A^{D}\right)}^{T}{\left(A^{D}{\left(P^{D}\right)}^{-1}{\left(A^{D}\right)}^{T}\right)}^{-1}\delta L^{D} \end{array}\right.$$

Finally, the optimized results of the observed values are obtained:

(17) $${\overset{̑}{X}}^{D}=X^{M}+\delta {\overset{̑}{X}}^{M}$$

The above optimized process for obtaining local common point coordinates is summarized, and the pseudo-code is provided in Algorithm 1.

**Algorithm 1**: Distance-constraint-based optimization for local common point coordinates
**Input:**

Initial coordinates of observation points $X^{M}$

**Output:**

Coordinates correction data of observation points $\delta {\overset{̑}{X}}^{M}$

1Compute coordinate corrections $\delta {\overset{̑}{X}}^{M}$ and optimized coordinates ${\overset{̑}{X}}^{M}$

2**For** *i* = 2 to *n* **do**
3   **For** *j* = *I* − 1 **do**
4Build the measurement equation $f_{D}$ of the distance observation (Equation (9));
5Build the residual equation $A^{D}\delta {\overset{̑}{X}}^{M}-\delta L^{D}=0$ (Equation (12));
6**End for**;
7**End for**;
8**Do begin**;
9Build the objective function $\Phi^{D}$ (Equation (13));
10Calculate the minimum value $\Phi^{D}$ for $\delta {\overset{̑}{X}}^{M}$ (Equation (14));
11Set $\frac{d\Phi^{D}}{d\left(\delta {\overset{̑}{X}}^{M}\right)}={\left(\delta {\overset{̑}{X}}^{M}\right)}^{T}P^{D}-{\left(K^{D}\right)}^{T}A^{D}=0$;
12Compute the contact number vector $K^{D}$ (Equation (15));
13**Return** 
$\delta {\overset{̑}{X}}^{M}$

14**Set** 
${\overset{̑}{X}}^{D}=X^{M}+\delta {\overset{̑}{X}}^{M}$


### 3.4. Correction Model for Coordinate Transformation Parameters Considering Multi-Source Heterogeneous Measurement Error Compensation

During the estimation of reference frame transformation parameters, challenges arise due to the limited field of view of the 3D scanner and the dense distribution of local common points on the MNCT, which increases the correlation among those points. Moreover, large-angle rotations often lead to instability in the solution of transformation parameters, and the accumulation of multi-source heterogeneous measurement errors further degrades the accuracy and reliability of the computed results. Therefore, based on the above error optimization strategy for coordinate measurement values, this section constructs a transformation parameter correction model that takes into account the compensation for multi-source heterogeneous measurement errors.

**Figure 7:**
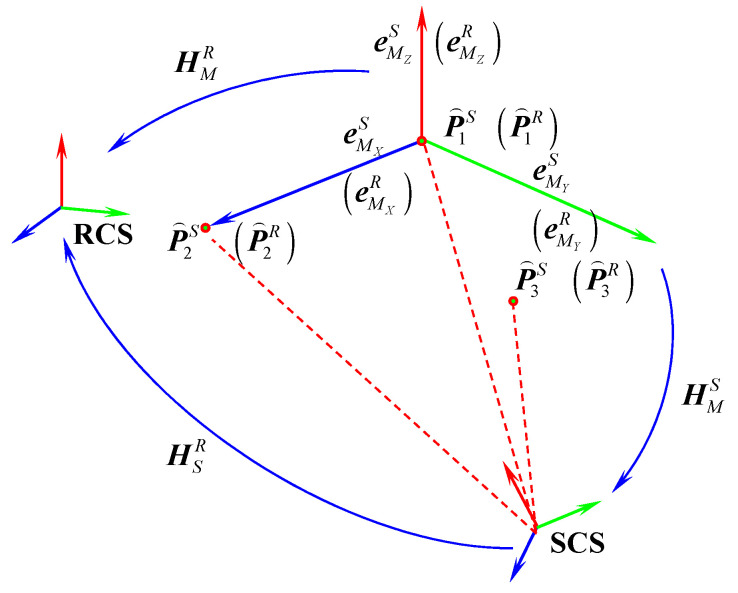
Schematic diagram for solving coordinate system transformation parameters.

As an example, the estimation of the transformation matrix $H_{S}^{R}$ parameters between the SCS and RCS is carried out using a stepwise strategy while accounting for arbitrary rotation angles, as illustrated in Figure 7. Specifically, this strategy begins by applying the coordinate optimization method introduced in Section 3.3 to compensate for measurement errors of local common points. Subsequently, a middle reference frame is constructed using three non-collinear points to derive an initial estimate of the transformation parameters. Finally, the parameters are refined using the Bursa–Wolf model combined with a weighted least-squares criterion, resulting in improved accuracy of the final solution.

Let ${\overset{̑}{P}}_{1}^{S}$, ${\overset{̑}{P}}_{2}^{S}$, and ${\overset{̑}{P}}_{3}^{S}$ denote the compensated coordinates of three non-collinear points on the MNCT in the SCS, and let ${\overset{̑}{P}}_{1}^{R}$, ${\overset{̑}{P}}_{2}^{R}$, and ${\overset{̑}{P}}_{3}^{R}$ represent their corresponding compensated coordinates in the RCS. Using point ${\overset{̑}{P}}_{1}^{S}$ as the origin, a transitional coordinate frame ${\overset{̑}{P}}_{1}^{S}e_{M_{X}}^{S}e_{M_{Y}}^{S}e_{M_{Z}}^{S}$ is constructed via the three-point method. Based on this, the transformation matrix from the SCS to the middle transitional coordinate system (MCS) can be derived as follows:

(18) $$\left\{\begin{array}{l} H_{M}^{S}=\left[\begin{array}{cc} R_{M}^{S} & T_{M}^{S}\\ 0 & 1 \end{array}\right]\\ R_{M}^{S}=\left[\begin{array}{ccc} e_{M_{X}}^{S} & e_{M_{Y}}^{S} & e_{M_{Z}}^{S} \end{array}\right],\;T_{M}^{S}={\overset{̑}{P}}_{1}^{S} \end{array}\right.$$

where $e_{M_{X}}^{S}$, $e_{M_{Y}}^{S}$ and $e_{M_{Z}}^{S}$ are unit direction vectors.

Similarly, by establishing a transitional coordinate system centered at point ${\overset{̑}{P}}_{1}^{R}$, the transformation matrix of the MCS relative to RCS can be obtained as:

(19) $$\left\{\begin{array}{l} H_{M}^{R}=\left[\begin{array}{cc} R_{M}^{R} & T_{M}^{R}\\ 0 & 1 \end{array}\right]\\ R_{M}^{R}=\left[\begin{array}{ccc} e_{M_{X}}^{R} & e_{M_{Y}}^{R} & e_{M_{Z}}^{R} \end{array}\right],\;T_{M}^{R}={\overset{̑}{P}}_{1}^{R} \end{array}\right.$$

By combining Equations (18) and (19), the initial transformation matrix between the SCS and the RCS is obtained as follows:

(20) $${\overset{̑}{H}}_{S}^{R}=\left[\begin{array}{cc} {\overset{̑}{R}}_{S}^{R} & {\overset{̑}{T}}_{S}^{R}\\ 0 & 1 \end{array}\right]=\left[\begin{array}{cc} R_{M}^{R} & T_{M}^{R}\\ 0 & 1 \end{array}\right]{\left[\begin{array}{cc} R_{M}^{S} & T_{M}^{S}\\ 0 & 1 \end{array}\right]}^{-1}$$

where ${\overset{̑}{t}}_{Sx}^{R}$, ${\overset{̑}{t}}_{Sy}^{R}$ and ${\overset{̑}{t}}_{Sz}^{R}$ represent the corresponding initial translation parameters, while ${\overset{̑}{w}}_{Sx}^{R}$, ${\overset{̑}{w}}_{Sy}^{R}$ and ${\overset{̑}{w}}_{Sz}^{R}$ represent the initial rotation angle parameters:

(21) $$\left\{\begin{array}{l} {\overset{̑}{w}}_{Sx}^{R}=\arctan\left({\overset{̑}{R}}_{S}^{R}\left(3,\;2\right)/{\overset{̑}{R}}_{S}^{R}\left(3,\;3\right)\right)\\ {\overset{̑}{w}}_{Sy}^{R}=-\arcsin\left({\overset{̑}{R}}_{S}^{R}\left(3,\;2\right)\right)\\ {\overset{̑}{w}}_{Sz}^{R}=\arctan\left({\overset{̑}{R}}_{S}^{R}\left(2,\;1\right)/{\overset{̑}{R}}_{S}^{R}\left(1,\;1\right)\right) \end{array}\right.$$

where ${\overset{̑}{R}}_{S}^{R}\left(i,\;j\right)$ represents the element in the *i*-th row and *j*-th column of the rotation matrix ${\overset{̑}{R}}_{S}^{R}$.

Let $P_{i}^{S}$ be the measured coordinates of a common point in the SCS, and let ${\overset{̑}{P}}_{Si}^{R}=\left({\overset{̑}{x}}_{Si}^{R},\;{\overset{̑}{y}}_{Si}^{R},\;{\overset{̑}{z}}_{Si}^{R}\right)$ be the coordinates after the initial transformation. The relationship between them can be computed using the following equation:

(22) $${\overset{̑}{P}}_{Si}^{R}={\overset{̑}{R}}_{S}^{R}P_{i}^{S}+{\overset{̑}{T}}_{S}^{R}$$

At this stage, the reference transformation angle between $P_{i}^{R}$ and ${\overset{̑}{P}}_{Si}^{R}$ satisfies the small-angle condition. According to the Bursa–Wolf model, the transformation relationship between them is established as follows:

(23) $$P_{i}^{R}={\overset{̑}{P}}_{Si}^{R}+\Delta R_{S}^{R}{\overset{̑}{P}}_{Si}^{R}+\Delta T_{S}^{R}$$

where the specific forms of $\Delta R_{S}^{R}$ and $\Delta T_{S}^{R}$ are as follows:

(24) $$\left\{\begin{array}{l} \Delta R_{S}^{R}=\left[\begin{array}{ccc} \Delta u_{S}^{R} & -\Delta w_{Sz}^{R} & \Delta w_{Sy}^{R}\\ \Delta w_{Sz}^{R} & \Delta u_{S}^{R} & -\Delta w_{Sx}^{R}\\ -\Delta w_{Sy}^{R} & \Delta w_{Sx}^{R} & \Delta u_{S}^{R} \end{array}\right]\\ \Delta T_{S}^{R}={\left[\begin{array}{ccc} \Delta t_{Sx}^{R} & \Delta t_{Sy}^{R} & \Delta t_{Sz}^{R} \end{array}\right]}^{T} \end{array}\right.$$

where $\Delta u_{S}^{R}$ is the scaling factor; $\Delta w_{Sx}^{R}$, $\Delta w_{Sy}^{R}$ and $\Delta w_{Sz}^{R}$ are the rotational parameter errors based on the initial transformation, while $\Delta t_{Sx}^{R}$, $\Delta t_{Sy}^{R}$ and $\Delta t_{Sz}^{R}$ are the translational parameter errors.

Due to the existence of multi-source heterogeneous measurement errors, the corrected model of Equation (23) is obtained:

(25) $$P_{i}^{R}+\Delta P_{i}^{R}=\left({\overset{̑}{P}}_{Si}^{R}+\Delta {\overset{̑}{P}}_{Si}^{R}\right)+\Delta R_{S}^{R}\left({\overset{̑}{P}}_{Si}^{R}+\Delta {\overset{̑}{P}}_{Si}^{R}\right)+\Delta T_{S}^{R}$$

where $\Delta P_{i}^{R}=\left(\Delta x_{i}^{R},\;\Delta y_{i}^{R},\;\Delta z_{i}^{R}\right)$ represents the actual measurement error of the point obtained using the laser tracker, while $\Delta {\overset{̑}{P}}_{Si}^{R}=\left(\Delta {\overset{̑}{x}}_{Si}^{R},\;\Delta {\overset{̑}{y}}_{Si}^{R},\;\Delta {\overset{̑}{z}}_{Si}^{R}\right)$ refers to the initial transformation value of the measurement error for the 3D scanner-acquired point.

By omitting higher-order terms, the equation is further simplified as follows:

(26) $$P_{i}^{R}-{\overset{̑}{P}}_{Si}^{R}+\Delta P_{i}^{R}=\Delta R_{S}^{R}{\overset{̑}{P}}_{Si}^{R}+\Delta T_{S}^{R}+\Delta {\overset{̑}{P}}_{Si}^{R}$$

For each local common point, Equation (26) can be extended accordingly:

(27) $$\underset{l_{S}^{R}}{\underset{︸}{\left(\begin{array}{c} x_{1}^{R}-{\overset{̑}{x}}_{S1}^{R}\\ y_{1}^{R}-{\overset{̑}{y}}_{S1}^{R}\\ z_{1}^{R}-{\overset{̑}{z}}_{S1}^{R}\\ \vdots \\ x_{n}^{R}-{\overset{̑}{x}}_{Sn}^{R}\\ y_{n}^{R}-{\overset{̑}{y}}_{Sn}^{R}\\ z_{n}^{R}-{\overset{̑}{z}}_{Sn}^{R} \end{array}\right)}}+\underset{V_{\Pi }}{\underset{︸}{\left(\begin{array}{c} \Delta x_{1}^{R}\\ \Delta y_{1}^{R}\\ \Delta z_{1}^{R}\\ \vdots \\ \Delta x_{n}^{R}\\ \Delta y_{n}^{R}\\ \Delta z_{n}^{R} \end{array}\right)}}=\underset{A_{S}^{R}}{\underset{︸}{\left(\begin{array}{ccccccc} 1 & 0 & 0 & 0 & {\overset{̑}{z}}_{S1}^{R} & -{\overset{̑}{y}}_{S1}^{R} & {\overset{̑}{x}}_{S1}^{R}\\ 0 & 1 & 0 & -{\overset{̑}{z}}_{S1}^{R} & 0 & {\overset{̑}{x}}_{S1}^{R} & {\overset{̑}{y}}_{S1}^{R}\\ 0 & 0 & 1 & {\overset{̑}{y}}_{S1}^{R} & {\overset{̑}{x}}_{S1}^{R} & 0 & {\overset{̑}{z}}_{S1}^{R}\\ \vdots & \vdots & \vdots & \vdots & \vdots & \vdots & \vdots \\ 1 & 0 & 0 & 0 & {\overset{̑}{z}}_{Sn}^{R} & -{\overset{̑}{y}}_{Sn}^{R} & {\overset{̑}{x}}_{Sn}^{R}\\ 0 & 1 & 0 & -{\overset{̑}{z}}_{Sn}^{R} & 0 & {\overset{̑}{x}}_{Sn}^{R} & {\overset{̑}{y}}_{Sn}^{R}\\ 0 & 0 & 1 & {\overset{̑}{y}}_{Sn}^{R} & {\overset{̑}{x}}_{Sn}^{R} & 0 & {\overset{̑}{z}}_{Sn}^{R} \end{array}\right)}}\underset{\Delta \xi_{S}^{R}}{\underset{︸}{\left(\begin{array}{c} \Delta w_{Sx}^{R}\\ \Delta w_{Sy}^{R}\\ \Delta w_{Sz}^{R}\\ \Delta t_{Sx}^{R}\\ \Delta t_{Sy}^{R}\\ \Delta t_{Sz}^{R}\\ \Delta \mu_{S}^{R} \end{array}\right)}}+\underset{V_{Ι}}{\underset{︸}{\left(\begin{array}{c} \Delta {\overset{̑}{x}}_{S1}^{R}\\ \Delta {\overset{̑}{y}}_{S1}^{R}\\ \Delta {\overset{̑}{z}}_{S1}^{R}\\ \vdots \\ \Delta {\overset{̑}{x}}_{Sn}^{R}\\ \Delta {\overset{̑}{y}}_{Sn}^{R}\\ \Delta {\overset{̑}{z}}_{Sn}^{R} \end{array}\right)}}$$

where $l_{S}^{R}$ denotes the difference vector between the local common point coordinates and the transformed measured coordinates in the RCS; $A_{S}^{R}$ is the coefficient matrix; $\Delta \xi_{S}^{R}$ represents the vector of transformation parameter errors to be solved; $V_{\Pi }$ is the measurement error vector obtained from the laser tracker; and $V_{Ι}$ denotes the vector of the values obtained after the initial transformation of the measurement error values of the scanner.

Let $V=V_{\Pi }-V_{Ι}$, then the error equation can be expressed as follows:

(28) $$V=A_{S}^{R}\Delta \xi_{S}^{R}-l_{S}^{R}$$

The above equation is a classic indirect adjustment model. Obviously, the measurement errors of laser trackers and scanners are independent of each other and follow the principle of weighted sum of squares minimum:

(29) $${\left(V_{\Pi }\right)}^{T}{\left(D_{\Pi }\right)}^{-1}V_{\Pi }+{\left(V_{Ι}\right)}^{T}{\left(D_{Ι}\right)}^{-1}V_{Ι}=\min$$

where $D_{\Pi }$ represents the variance–covariance matrix of the measurement errors for the local common point coordinates in the RCS, which can be calculated using the method described in reference [25]; $D_{Ι}$ is the variance–covariance matrix of measurement errors for the local common point coordinates after the initial coordinate transformation in the SCS:

(30) $$D_{Ι}={\overset{̑}{R}}_{S}^{R}D_{S}{\left({\overset{̑}{R}}_{S}^{R}\right)}^{T}$$

where $D_{S}$ is the variance matrix of the scanner measurement error:

(31) $$D_{S}=\left[\begin{array}{ccc} \sigma_{sx}^{2} & 0 & 0\\ 0 & \sigma_{sy}^{2} & 0\\ 0 & 0 & \sigma_{sz}^{2} \end{array}\right]$$

where $\sigma_{sx}^{2}$, $\sigma_{sy}^{2}$ and $\sigma_{sz}^{2}$ are the variances of the coordinate measurement errors of the scanner in the X, Y, and Z directions, respectively.

The objective function is formulated using the Lagrange extremum method as follows:

(32) $$\Phi ={\left(V_{\Pi }\right)}^{T}{\left(D_{\Pi }\right)}^{-1}V_{\Pi }+{\left(V_{Ι}\right)}^{T}{\left(D_{Ι}\right)}^{-1}V_{Ι}-2{\left(K_{S}^{R}\right)}^{T}\left(l_{S}^{R}+V_{\Pi }-V_{Ι}-A_{S}^{R}\Delta {\xi V}_{S}^{R}\right)$$

where ${\left(K_{S}^{R}\right)}^{T}$ is the contact vector; By taking the partial derivatives of $V_{Ι}$, $V_{\Pi }$ and $\Delta \xi_{S}^{R}$ with respect to their respective parameters and setting them to zero, the following result is obtained:

(33) $$\left\{\begin{array}{l} \frac{\partial \Phi }{\partial V_{\Pi }}=2{\left(V_{\Pi }\right)}^{T}{\left(D_{\Pi }\right)}^{-1}-2{\left(K_{S}^{R}\right)}^{T}=0\\ \frac{\partial \Phi }{\partial V_{Ι}}=2{\left(V_{Ι}\right)}^{T}{\left(D_{Ι}\right)}^{-1}+2{\left(K_{S}^{R}\right)}^{T}=0\\ \frac{\partial \Phi }{\partial \left(\Delta \xi_{S}^{R}\right)}=2{\left(K_{S}^{R}\right)}^{T}A_{S}^{R}=0 \end{array}\right.$$

Then, we obtain:

(34) $$K_{S}^{R}={\left(D_{Ι}+D_{\Pi }\right)}^{-1}\left(A_{S}^{R}\Delta \xi_{S}^{R}-l_{S}^{R}\right)$$

Thus, the least-squares solution of $\Delta \xi_{S}^{R}$ can be expressed as:

(35) $$\Delta \xi_{S}^{R}={\left({\left(A_{S}^{R}\right)}^{T}{\left(D_{Ι}+D_{\Pi }\right)}^{-1}A_{S}^{R}\right)}^{-1}{\left(A_{S}^{R}\right)}^{T}{\left(D_{Ι}+D_{\Pi }\right)}^{-1}l_{S}^{R}$$

Finally, by incorporating the initial reference transformation parameters, the optimized transformation matrix ${\tilde{H}}_{S}^{R}$ is obtained. Similarly, the optimized transformation matrix ${\tilde{H}}_{I}^{R}$ is derived. Based on Equation (7), the optimized calibration matrix ${\tilde{H}}_{S}^{I}$ is then calculated.

## 4. Construction of Large-Scale Spatial Measurement Field

**Figure 8:**
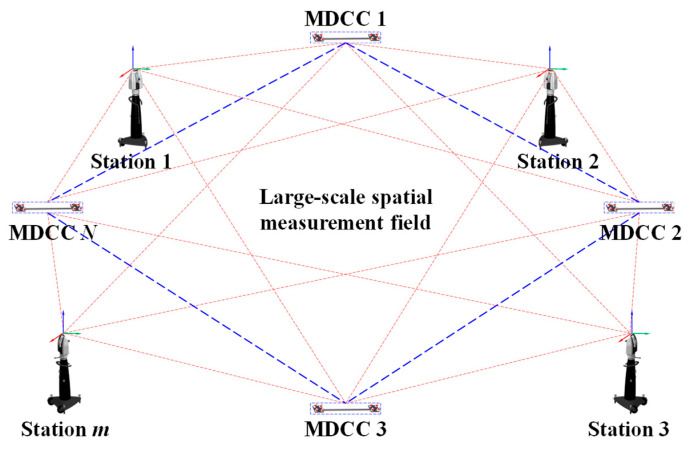
Schematic diagram of large-scale spatial measurement field construction with hierarchical measurement error control.

To address the issue of effectively controlling measurement errors of GCPs during the construction of large-scale spatial measurement fields for global calibration under constrained layouts, this section proposes an optimization method for coordinate measurement values considering the hierarchical measurement error control, which further improved the position measurement accuracy of GCPs across the entire measurement range. As illustrated in Figure 8, the principle of the proposed method is schematically shown.

**Figure 9:**
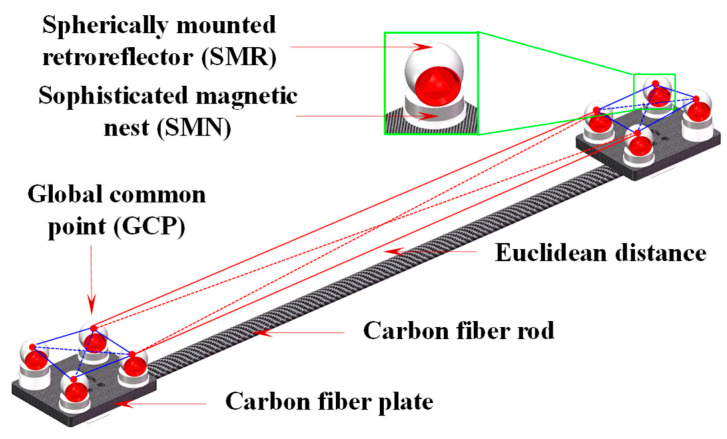
Diagram of Euclidean distances in the MDCC.

First, to eliminate gross errors in the measurement of GCPs and to monitor the working performance of the laser tracker on-site, thereby ensuring measurement consistency, a distance-based model was developed to reject out-of-tolerance measurement points. A multi-dimensional cooperative calibrator (MDCC) based on the four-point non-coplanar principle is introduced, consisting of carbon fiber plates, carbon fiber rods, SMNs, and SMRs, as shown in Figure 9. By leveraging the characteristic that the distance between any two points is independent of the coordinate system, the geometric relationship between the target points on the MDCC is calibrated using a high-precision CMM, thereby constructing multiple posture distance constraints. The laser tracker acquires measurements of the target points on the MDCC, which serve as GCPs. By calculating the difference between the measured and calibrated distances between two GCPs and comparing this difference with the allowable error limit, it can determine whether the measurement value of the GCP is out of tolerance.

Let $\left(x_{i}^{k},\;y_{i}^{k},\;z_{i}^{k}\right)$ and $\left(x_{j}^{k},\;y_{j}^{k},\;\right.\left.z_{j}^{k}\right)$ represent the measured coordinates of two points used to construct the spatial constraint distance, as measured by the laser tracker at station $k\left(k=1,\;2,\;\cdots ,\;m\right)$. Then, the following equation can be established:

(36) $${\left(L_{ij}^{k}\right)}^{2}={\left(L_{ij}+\Delta L_{ij}^{k}\right)}^{2}={\left(x_{i}^{k}-x_{j}^{k}\right)}^{2}+{\left(y_{i}^{k}-y_{j}^{k}\right)}^{2}+{\left(z_{i}^{k}-z_{j}^{k}\right)}^{2}\left(i\neq j\right)$$

where $L_{ij}^{k}$ denotes the measured spatial constraint distance, $L_{ij}$ represents the nominal distance between the two points, and $\Delta L_{ij}^{k}$ is the difference between the measured and nominal distance.

The allowable tolerance for each constraint distance is defined according to the measured distance and the required measurement accuracy:

(37) $$\Delta_{\lim}\geq \Delta L_{ij}^{k}$$

where $\Delta_{\lim}$ is the allowable tolerance.

In the measured data at the site, if the measurement error of a certain distance on the MDCC exceeds the allowable error limit, it can be judged that one of the two associated points has a significant error, indicating the presence of an unqualified point. Conversely, if the distances from a given target point to all other target points fall within the specified tolerance range, the point can be considered a qualified GCP. After obtaining the qualified GCPs, further control of their measurement errors is required. In this study, the large-scale spatial measurement field, established through multi-station laser tracker measurements combined with multiple MDCCs, essentially forms an edge-type measurement network. During the adjustment stage, the precision with which the initial GCP coordinates are assigned plays a decisive role in the accuracy of the final coordinate transformation parameters.

To enhance the assignment accuracy of the initial coordinates of both the laser tracker stations and the GCPs, this section first employs the 7-parameter Procrustes method to establish the preliminary orientation of multiple stations. Subsequently, a measurement model is developed for each station to obtain the corresponding covariance and Jacobian matrices. Following the matrix-weighted linear minimum variance fusion criterion, the weight matrix is derived to effectively fuse the multi-station measurement data and estimate the 3D coordinates of the GCPs. Finally, based on the common point transformation method, the initial 3D coordinates of the laser tracker stations are determined. Building on this foundation, the high-precision interferometric distance measurements of the laser tracker, combined with multi-domain and multi-pose length constraints, are utilized to further optimize the 3D coordinates of the GCPs. Through this procedure, the impact of angular measurement errors on spatial point localization is mitigated, thereby enabling refined correction of GCP measurement deviations.

Let $\left(X_{k},\;Y_{k},\;Z_{k}\right)$ denote the measurement station coordinates of the *k*-th $\left(k=1,\;2,\;\cdots ,\;m\right)$ laser tracker, and let $\left(x_{i},\;y_{i},\;z_{i}\right)$ denote the coordinates of the *i*-th $\left(i=1,\;2,\;\cdots ,\;n\right)$ GCP. Based on the distance formula, the corresponding error equation is established as follows:

(38) $${\left(l_{ik}+v_{ik}\right)}^{2}={\left(x_{i}-X_{k}\right)}^{2}+{\left(y_{i}-Y_{k}\right)}^{2}+{\left(z_{i}-Z_{k}\right)}^{2}$$

where $l_{ik}$ represents the laser interferometric measurement length value of the laser tracker at the *k*-th station for the *i*-th common point, and $v_{ik}$ represents the corresponding error.

By linearizing Equation (38), the following expression is obtained:

(39) $$v_{ik}=a_{ik}\left(\Delta X_{k}-\Delta x_{i}\right)+b_{ik}\left(\Delta Y_{k}-\Delta y_{i}\right)+c_{ik}\left(\Delta Z_{k}-\Delta z_{i}\right)+l_{ik}^{0}-l_{ik}$$

where $\left(\Delta X_{k},\;\Delta Y_{k},\;\Delta Z_{k}\right)$ and $\left(\Delta x_{i},\;\Delta y_{i},\;\Delta z_{i}\right)$ represent the correction values for the measurement station center coordinates and the GCP coordinates, respectively; $l_{ik}^{0}$ is the oblique distance computed from the initial coordinate values, and $a_{ik}$, $b_{ik}$, and $c_{ik}$ are the corresponding coefficients.

Based on the above analysis, *n* constraint equations can be formulated under a single laser tracker measurement station. Therefore, for *m* independent measurement stations, a total of *m* × *n* equations can be established. Equation (39) can thus be rewritten in matrix form as follows:

(40) $$V^{G}=A^{G}\Delta X^{G}-b^{G}$$

where $V^{G}={\left[v_{11},\cdots ,v_{mn}\right]}^{T}$, $b^{G}={\left[l_{11}-l_{11}^{0},\cdots ,l_{mn}-l_{mn}^{0}\right]}^{T}$; Vector $\Delta X^{G}={\left[\Delta X_{1},\cdots ,\Delta z_{m+n}\right]}^{T}$ consists of the correction terms for both the measurement station coordinates and the measured coordinates of the common points, while $A^{G}$ denotes the corresponding coefficient matrix.

In Equation (40), the unknown parameters to be estimated are the 3D correction values of both the GCPs and the laser tracker measurement stations. The total number of unknown parameters is 3m + 3n, and the number of equations in the error equation system is *m* × *n*. Due to the rank deficiency of the system matrix $A^{G}$, a unique solution can be obtained by introducing centroid-based reference constraints in accordance with the principles of rank-deficient network adjustment. However, this approach may lead to uneven distribution of point accuracy across the measurement field. To address this issue, based on the previously established error model, in this section, by combining the multi-region and multi-attitude length constraint conditions in space, an optimization model for coordinate measurement values based on spatial multi-attitude length constraints is established:

(41) $$d_{ij}\Delta x_{j}+e_{ij}\Delta y_{j}+f_{ij}\Delta z_{j}-d_{ij}\Delta x_{i}+e_{ij}\Delta y_{i}+f_{ij}\Delta z_{i}+w_{ij}=0$$

where vectors $\left(\Delta x_{i},\;\Delta y_{i},\;\Delta z_{i}\right)$ and $\left(\Delta x_{j},\;\Delta y_{j},\;\Delta z_{j}\right)$ represent the coordinate corrections of the constrained points, while $d_{ij}$, $e_{ij}$, and $f_{ij}$ denote the associated coefficient matrices, and $w_{ij}$ is the constant term. By combining Equations (40) and (41), the following system can be obtained:

(42) $$\left\{\begin{array}{c} V^{G}=A^{G}\Delta X^{G}-b^{G}\\ 0=B^{G}\Delta X^{G}+W_{x} \end{array}\right.$$

where $B^{G}$ is a computationally large sparse matrix composed of first-order derivative terms.

Under the constrained optimization method, the objective function is given by:

(43) $$\Phi^{G}={\left(V^{G}\right)}^{T}P_{\Delta X}V^{G}+2{\left(K^{G}\right)}^{T}(B^{G}\Delta X^{G}+W_{x})$$

where $K^{G}$ is the vector of association coefficients.

Finally, the correction values for coordinate optimization are obtained as:

(44) $$\Delta X^{G}=\left(N_{AA}^{-1}-N_{AA}^{-1}{\left(B^{G}\right)}^{T}N_{BB}^{-1}B^{G}N_{AA}^{-1}\right)W_{b}-N_{AA}^{-1}{\left(B^{G}\right)}^{T}N_{BB}^{-1}W_{x}$$

where $N_{AA}={\left(A^{G}\right)}^{T}P_{\Delta X}A^{G}$ is the invertible matrix, $W_{b}={\left(A^{G}\right)}^{T}P_{\Delta X}b^{G}$.

## 5. Experimental and Discussion

### 5.1. System Construction

**Figure 10:**
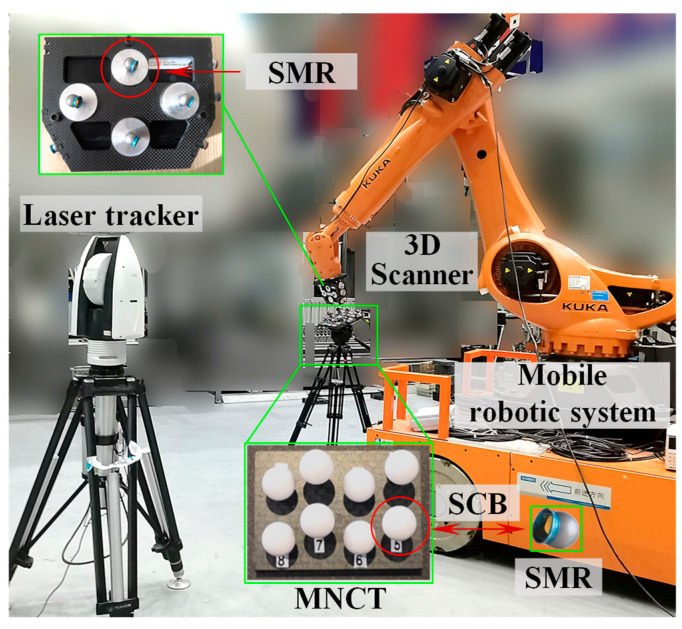
Overview of the robot-assisted laser scanning hybrid measurement system.

To validate the effectiveness of the proposed calibration method, a hybrid measurement system integrating global laser tracking and high-precision local scanning was constructed, and corresponding calibration experiments were conducted. As illustrated in Figure 10, the setup consists of a laser tracker, a structured-light 3D scanner, and a mobile robotic platform that integrates an industrial robot with an AGV. Specifically, the main structure of the MNCT is made of stable performance carbon fiber material, with eight SMNs for placing 0.5-inch target balls (SCB and SMR) on the surface, achieving a repeatability accuracy of up to 0.001 mm. The observation target of the laser tracker is the 0.5-inch SMR, and the spherical deviation is approximately 0.002 mm. The observation target of the 3D scanner is the 0.5-inch SCB, and the spherical deviation is approximately 0.0015 mm. Therefore, the center positions of these two types of observation targets have a high spatial co-location accuracy. Furthermore, several 0.5-inch SMNs were arranged on the surface of the 3D scanner to accommodate the 0.5-inch SMR, enabling the laser tracker to accurately locate and track the position and orientation of the 3D scanner.

The binocular structured-light 3D scanner used in this study was the LMI Gocator3 series (LMI Technologies Inc., Burnaby, BC, Canada), featuring a resolution of 5 million pixels, an X-Y spatial resolution of 0.025 mm, a measurement depth range of 87 mm, a field of view (FOV) of 27 mm × 45 mm, and a verified VDE accuracy of 0.025 mm. The laser tracker employed in this study was the Leica AT960-MR (Leica Geosystems AG, Heerbrugg, Switzerland), offering a full 360° horizontal rotation and a ±145° vertical tilt range. It features an angular resolution of 0.07 arcseconds, an angular accuracy of 1.7 arcseconds, and a maximum measurement range of 80 m. The full-range measurement accuracy is specified as ± (0.015 + 6 ppm × L) mm, where L represents the measured distance in meters. Prior to the experimental procedure, the spatial relationships among the centers of the target spheres in MDCCs and MNCT were calibrated in advance using a Zeiss PRISMO coordinate measuring machine (Carl Zeiss Industrielle Messtechnik GmbH, Oberkochen, Germany), ensuring high measurement accuracy and experimental reliability. For the typical distances between target spheres in our experiments, the resulting measurement error is less than 0.0015 mm. A conservative combined uncertainty of ±0.002 mm (k = 2) was determined by integrating Type A analysis from repeated measurements with Type B estimates derived from instrument specifications and environmental stability. The integrated uncertainty provides a reliable reference for evaluating the calibration accuracy of the developed method. All experimental procedures were performed under controlled laboratory conditions, where the ambient temperature was maintained within 22–23 °C and the relative humidity was kept at 55–60%.

### 5.2. Extrinsic Parameter Calibration Experiments

Firstly, the relative pose of the laser tracker, 3D scanner, and MNCT was varied to acquire several distinct measurement positions for the two instruments, while the MNCT itself was kept fixed. At each measurement position, both instruments repeatedly measured the respective observation target points on the MNCT, thereby generating redundant datasets. After noise reduction and centroid estimation of the scanning sphere (Section 3.2), the optimization procedure in Section 3.3 and Section 3.4 was applied to calculate refined and corrected target point coordinates of LCPs acquired by 3D scanner and laser tracker, as reported in Table 1 and Table 2.

In addition, the angle-constrained coordinate optimization method reported in reference [25] was applied to derive optimal and corrected coordinates for the scanning pose reference point, with results summarized in Table 3.

Based on the above data and in combination with the correction model for the reference transformation parameters, the optimized extrinsic calibration matrix ${\tilde{H}}_{S}^{I}$ is obtained as follows:

$${\tilde{H}}_{S}^{I}=\left[\begin{array}{cccc} 0.00312 & 0.00243 & -0.99999 & 214.23490\\ -0.06656 & -0.99777 & -0.00264 & 110.93797\\ -0.99777 & 0.06656 & -0.00295 & -7.73651\\ 0 & 0 & 0 & 1 \end{array}\right]$$

**Figure 11:**
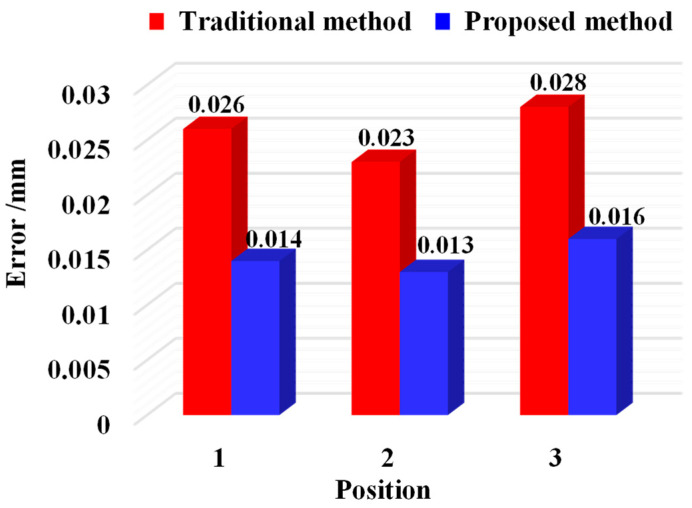
Mean calibration error at different locations.

To validate the calibration accuracy, the three-dimensional coordinates dispersion of local common points is calculated using the calibration matrix before and after optimization; that is, the calibration accuracy of extrinsic parameters is measured by the root mean square error of the distance between the two points before and after optimization. In this study, the traditional indirect calibration method and the calibration method proposed are used to calculate the calibration errors of the extrinsic parameters, respectively, and the mean calibration errors under multiple positions are obtained, as shown in Figure 11. The conventional calibration approach resulted in mean extrinsic parameter calibration errors of 0.026, 0.023, and 0.028 mm at Positions 1–3, whereas the distance-constrained method reduced these errors to 0.014, 0.013, and 0.016 mm, respectively.

The proposed distance-constrained method maintained a mean calibration error below 0.016 mm across all positions, outperforming the conventional indirect calibration, which showed a mean error of 0.028 mm, thereby confirming its effectiveness.

### 5.3. Experiments on the Construction of Large-Scale Spatial Measurement Field

**Figure 12:**
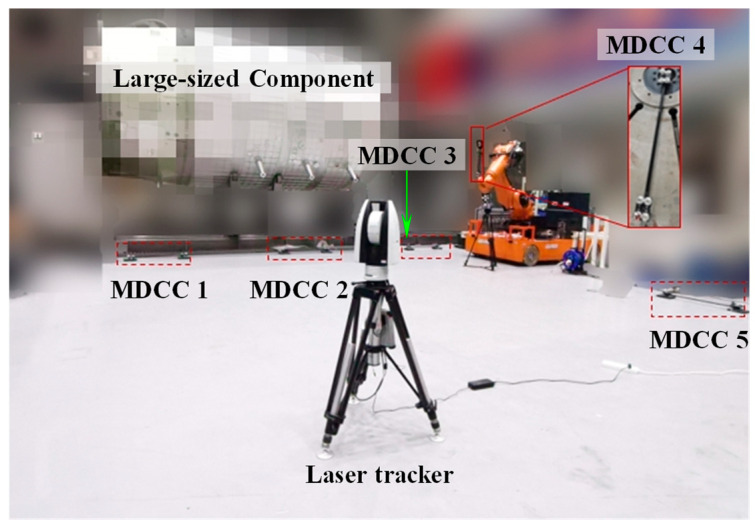
Construction experiment of large-scale spatial measurement field on-site.

**Figure 13:**
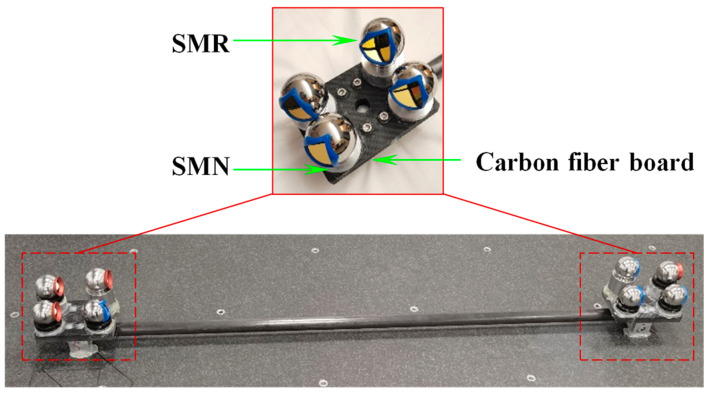
Multi-dimensional cooperative calibrator.

Following the method outlined in Section 4, a high-precision spatial measurement field was established over a 14 × 6 × 3 m volume, serving as a global reference framework for precise measurements. The experimental site configuration is illustrated in Figure 12. For accurate global calibration, several MDCCs were developed specifically. As shown in Figure 13, the MDCC is primarily constructed from carbon fiber and has an approximate length of 1000 mm. Eight 1.5-inch SMNs for 1.5-inch SMRs are rigidly attached along its surface. Before conducting the experiments, the spatial arrangements of all SMR centers were accurately calibrated with a high-precision CMM to guarantee reliable and precise experimental data.

Firstly, five MDCCs were deployed across the measurement site, with the maximum elevation difference between common points reaching approximately 3 m. Four measurement stations are set up at different locations. At each station, the laser tracker measured the GCPs on the MDCCs. The first station’s coordinate system served as the GCS, while the remaining three were treated as local coordinate systems for subsequent measurements.

Subsequently, based on the measured distance between the laser tracker and the common points, permissible error thresholds were defined to identify and exclude outliers on site. Once the gross errors were removed and qualified data were retained, the measured coordinates of the common points from the three LCSs were transformed into the GCS. Then, by adopting the method proposed in this study, the coordinate measurement values of the GCPs with redundant measurements are optimized. For each MDCC, two representative GCPs were selected. Applying the method presented in Section 4, optimized coordinates along with their associated correction values for the selected GCPs within the GCS and the LCS1 were determined, as summarized in Table 4 and Table 5.

Currently, evaluating the accuracy of large-scale spatial measurement networks often relies on computing station transfer errors of GCPs [4]. For this, three methods were applied: the best-fit algorithm in Spatial Analyzer 2023.1 [29,30], the single-distance constraint method, and the proposed method. The maximum registration (MPE) and mean registration errors (ME) of selected GCPs obtained from these methods are listed in Table 6.

**Figure 14:**
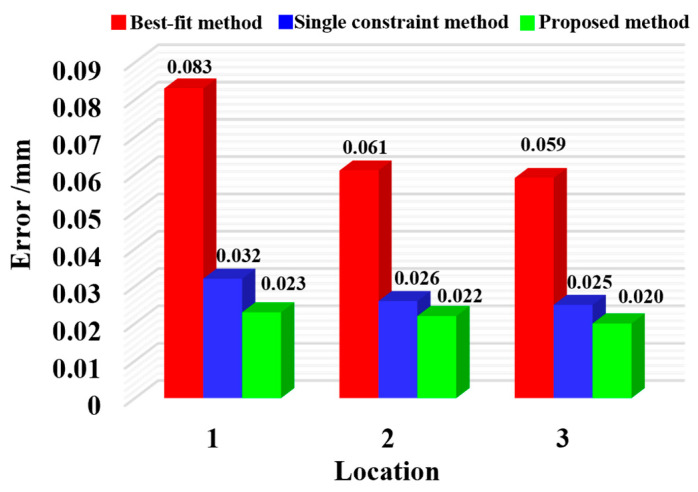
Mean registration error of all GCPs at each local measuring station.

In the GCS, the average station transfer error of the GCPs was calculated to be 0.078 mm using the best-fit method. In comparison, the single-distance constraint method reduced the error to 0.036 mm. Furthermore, the proposed method achieved an even lower average error of 0.033 mm, demonstrating improved accuracy in coordinate transformation. Furthermore, the average station transfer errors of all GCPs under each local measurement station were evaluated, as illustrated in Figure 14. Using the single-distance constraint method, the error magnitudes at local stations 1 through 3 decreased from 0.083, 0.061, and 0.059 mm to 0.032, 0.026, and 0.025 mm, respectively. Applying the method proposed in this study further reduced these errors to 0.023, 0.022, and 0.020 mm. Collectively, these results demonstrate the high effectiveness of the proposed calibration method.

### 5.4. Accuracy Verification and Analysis of the RLSHS

To ensure reliable measurement performance, a standardized and widely accepted accuracy verification method is essential for evaluating the measurement accuracy of RLSHS. There are generally two approaches for verifying measurement system accuracy. One involves using a higher-precision metrology device to evaluate the test results. However, this method has notable drawbacks: such devices are often costly, require experienced personnel for operation and maintenance, and demand significant time and resources for setup and calibration. Moreover, discrepancies in accuracy, measurement range, and system characteristics between different instruments can introduce uncertainties and compromise the consistency of the evaluation results. An alternative and more cost-effective method for accuracy evaluation involves using standard artifacts. In this method, the measurement system follows a predefined procedure to measure the calibrated reference object, and the results are then compared with the known pre-calibration values to assess measurement accuracy. Due to its practicality and lower cost, this method is more widely used for evaluating the accuracy of measurement systems.

**Figure 15:**
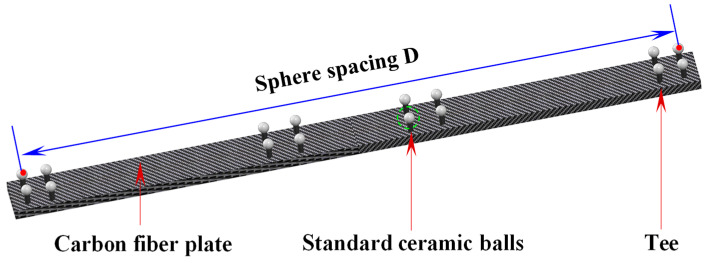
Diagram of sphere spacing in metric tool.

This study adopts the “sphere spacing error” (SSE) evaluation approach by measuring a calibrated metric tool, and the corresponding tests were performed in accordance with the international accuracy verification standard VDI/VDE 2634 Part 3 [31]. Sphere-to-sphere spacing error is commonly used as a primary indicator to assess measurement system accuracy. It is quantified as the deviation between the measured inter-sphere distance and the reference distance obtained from calibration. Evaluating this metric provides a comprehensive assessment of system performance and offers a reliable reference for ensuring overall measurement quality. To address considerations such as manufacturing complexity and cost, a customized metric tool was designed in this study. As illustrated in Figure 15, the metric tool consists of multiple spheres, corresponding holders, and a carbon fiber plate. The inter-sphere distances were precisely calibrated using a high-accuracy coordinate measuring machine.

**Figure 16:**
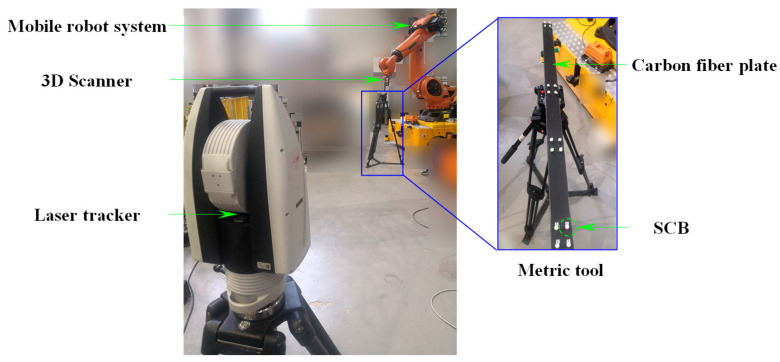
On-site accuracy verification for measurement system.

The accuracy validation procedure for the laser scanning hybrid measurement method is outlined as follows. First, a calibrated reference artifact mounted on an adjustable tripod is sequentially positioned at multiple heights and orientations throughout the measurement volume, while the laser tracker remains fixed. The mobile robot system transports the 3D scanner to various spatial locations, where the scanner captures the metric tool to assess measurement accuracy. Metric tool placements were configured to fully cover the measurement space. Since the sphere spacing exceeded the 3D scanner’s view, a multi-view strategy was employed to reconstruct sphere centers, while the laser tracker simultaneously captured reference points to determine the scanner’s position. All reconstructed sphere centers were then transformed from the SCS to the RCS. The reconstructed sphere centers were subsequently transformed into the global coordinate frame using the established large-scale spatial measurement field. Finally, the inter-sphere distance errors are computed for each metric tool position to evaluate the system’s measurement precision. The accuracy verification setup is illustrated in Figure 16.

Four spheres were selected from the metric tool as representatives, with calibrated inter-sphere distances of D1 = 851.476 mm and D2 = 1007.418 mm. Table 7 presents the measured inter-sphere distances along with their corresponding errors. For distance D1, the maximum error (MPE) and mean error (ME) were 0.113 mm and 0.108 mm, respectively, while for distance D2, these values were 0.117 mm and 0.112 mm. All measured errors are below the 0.2 mm threshold, indicating that the RLSHS developed using the proposed method meets the required accuracy standards.

### 5.5. Experimental Study and Analysis of On-Site Measurement for Key Geometrical Features of Large-Sized Components

**Figure 17:**
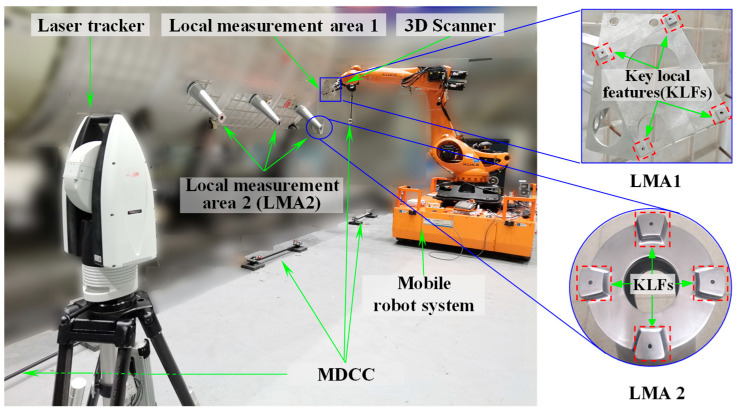
On-site measurement of key geometric features in LSC.

To validate the effectiveness and applicability of the proposed method, the RLSHS was deployed on-site for measurement of key geometrical features of large-sized components. On-site measurements were conducted on four key geometric features located on two typical local measurement areas (LMAs), as illustrated in Figure 17.

**Figure 18:**
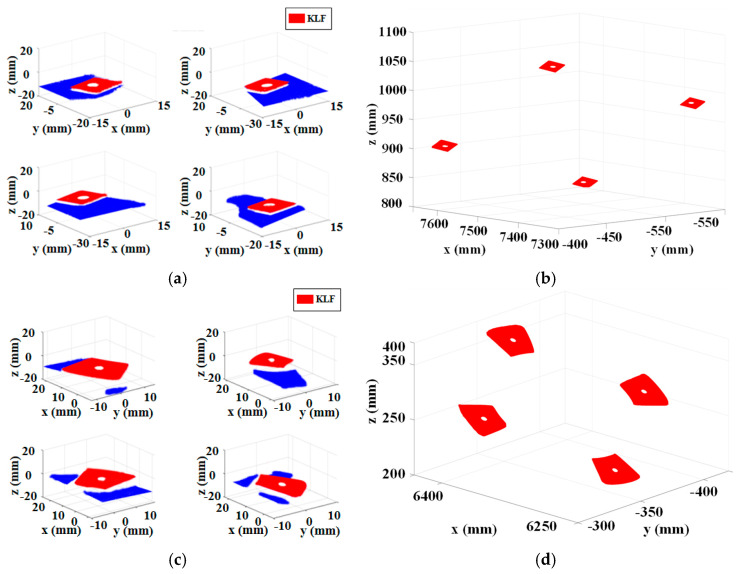
Scanning results of KLFs in LSC. (**a**) Point clouds of KLFs in LMA 1 at each station. (**b**) Reconstructed point clouds of KLFs in LMA 1. (**c**) Point clouds of KLFs in LMA 2 at each station. (**d**) Reconstructed point clouds of KLFs in LMA 2.

At the initial stage of measurement, the mobile platform first transports the industrial robot to a position near the target features. Subsequently, the scanner pose is established through laser tracker measurements of the SMRs fixed on the scanner. Then, the robot is used to position the 3D scanner at a series of predefined locations. At each location, the scanner captures the target area to generate the initial point cloud of the key geometric features, as illustrated in Figure 18a,c.

Subsequently, point cloud preprocessing was performed to eliminate noise and extract the target region, as illustrated in Figure 18b,d. The center coordinates of the holes in the SCS were subsequently derived based on the reconstruction method outlined in Section 3.2. Meanwhile, the laser tracker captured the reference observation point mounted on the 3D scanner to determine its current pose. Based on this pose, the computed hole centers were transformed from the SCS to the RCS. After completing the measurement of KLFs within the current workspace, the mobile robotic system advanced to the next station, where the same procedure was repeated until all target features had been inspected.

Finally, the three-dimensional global coordinates of the hole centers for all KLFs were obtained, as summarized in Table 8. The proposed RLSHS completed the inspection of the KLFs in approximately 8 min, whereas manual measurement required about 30 min for the same task. Quantitative comparative experiments indicate that the proposed measurement system and methodology significantly outperform traditional manual measurement in terms of time efficiency and measurement stability. Moreover, our experimental observations indicate that the system exhibits good robustness within moderate thermal fluctuations (approximately ±5–10 °C) typically encountered in industrial workshops. Overall, the developed method demonstrates strong applicability and provides reliable and effective measurement data to support high-quality manufacturing of large-scale components.

## 6. Conclusions

In this study, a robot-assisted laser scanning hybrid measurement method was developed to achieve accurate and automated 3D shape measurement of large-sized components with numerous small key local features. The proposed method integrates a laser tracker, a 3D scanner, and a mobile robotic system, thereby enabling flexible large-scale measurements. An accuracy-enhanced calibration strategy was introduced, including an accurate extrinsic parameter calibration method based on robust target sphere center estimation and distance-constrained optimization of local common points, as well as an improved global calibration method incorporating coordinate measurement value optimization and hierarchical error control. The accuracy validation experiments demonstrated that, over a measurement span of 14 m, the maximum error (MPE) reached 0.117 mm, while the mean error (ME) was 0.112 mm, thereby verifying the calibration method’s high precision. Moreover, large-scale scanning experiments conducted on representative local measurement areas of a complex large-sized component further verified the method’s applicability and its ability to provide reliable measurement data for high-quality manufacturing of large-sized components.

Future work will focus on developing advanced error modeling and compensation strategies to address environmental influences and robot-induced disturbances, such as thermal drift, vibration, and long-term stability issues, while also conducting a detailed analysis of the spatial distribution of measurement errors, including potential accuracy attenuation in edge regions. In addition, intelligent measurement planning methods, such as adaptive scanning and path optimization, will be further investigated to improve measurement efficiency and ensure comprehensive coverage of complex geometries.

## Figures and Tables

**Table 1:** Optimized coordinates and correction data of LCPs acquired by 3D scanner on the MNCT.

No.	${\tilde{x}}_{si}^{S}$/mm	${\tilde{y}}_{si}^{S}$/mm	${\tilde{z}}_{si}^{S}$/mm	$\Delta {\tilde{x}}_{si}^{S}$/mm	$\Delta {\tilde{y}}_{si}^{S}$/mm	$\Delta {\tilde{z}}_{si}^{S}$/mm
1	−6.107	−17.248	−9.555	−0.002	0.008	−0.006
2	−9.334	−6.339	−3.661	0.007	−0.004	−0.005
3	−5.467	5.741	−9.780	0.012	0.002	−0.004
4	9.322	5.707	−10.260	−0.015	0.003	−0.007
5	5.459	−6.167	−4.115	0.003	0.004	0.001
6	9.504	−17.260	−10.051	−0.005	0.003	−0.005

**Table 2:** Optimized coordinates and correction data of LCPs acquired by laser tracker on the MNCT.

No.	${\tilde{x}}_{gi}^{R}$/mm	${\tilde{y}}_{gi}^{R}$/mm	${\tilde{z}}_{gi}^{R}$/mm	$\Delta {\tilde{x}}_{gi}^{R}$/mm	$\Delta {\tilde{y}}_{gi}^{R}$/mm	$\Delta {\tilde{z}}_{gi}^{R}$/mm
1	4439.619	−1432.338	−199.469	−0.006	−0.002	0.002
2	4430.278	−1438.580	−193.311	0.003	0.008	−0.007
3	4419.383	−1443.164	−200.970	−0.004	0.003	−0.005
4	4412.970	−1431.178	−206.820	−0.005	−0.013	0.003
5	4423.679	−1426.684	−199.152	0.004	0.011	−0.006
6	4432.830	−1419.695	−205.640	−0.007	−0.008	0.001

**Table 3:** Optimized coordinates and correction data of scan position points acquired by laser tracker.

No.	${\tilde{x}}_{Ii}^{R}$/mm	${\tilde{y}}_{Ii}^{R}$/mm	${\tilde{z}}_{Ii}^{R}$/mm	$\Delta {\tilde{x}}_{Ii}^{R}$/mm	$\Delta {\tilde{y}}_{Ii}^{R}$/mm	$\Delta {\tilde{z}}_{Ii}^{R}$/mm
1	4276.124	−1423.866	1.565	0.003	−0.004	−0.021
2	4285.499	−1436.884	−39.293	0.002	−0.005	0.004
3	4314.775	−1446.752	−94.019	0.004	−0.007	−0.015
4	4314.427	−1372.497	−60.148	0.001	0.003	−0.019

**Table 4:** Optimized coordinates and correction data of partial GCPs in GCS.

No.	${\overset{̑}{x}}_{i}^{G}$/mm	$\Delta x_{i}^{G}$/mm	${\overset{̑}{y}}_{i}^{G}$/mm	$\Delta y_{i}^{G}$/mm	${\overset{̑}{z}}_{i}^{G}$/mm	$\Delta z_{i}^{G}$/mm
1	1577.987	−0.049	6629.338	0.018	−1223.005	−0.109
2	3696.428	0.051	4429.587	−0.020	−1225.259	−0.017
3	4319.382	0.059	3617.086	−0.027	−1215.178	0.050
4	6461.415	0.017	1197.048	−0.042	−1231.484	−0.045
5	1843.496	−0.066	−2553.324	−0.040	−1263.557	−0.061
6	1097.620	−0.077	−3226.831	−0.050	−1283.210	0.047
7	9354.485	0.068	228.403	0.067	663.813	0.098
8	9394.815	−0.016	263.707	0.044	1671.130	0.136

**Table 5:** Optimized coordinates and correction data of partial GCPs in LCS1.

No.	${\overset{̑}{x}}_{i}^{L_{1}}$/mm	$\Delta x_{i}^{L_{1}}$/mm	${\overset{̑}{y}}_{i}^{L_{1}}$/mm	$\Delta y_{i}^{L_{1}}$/mm	${\overset{̑}{z}}_{i}^{L_{1}}$/mm	$\Delta z_{i}^{L_{1}}$/mm
1	4999.718	−0.013	6352.989	−0.005	−1087.086	−0.106
2	7589.819	−0.019	4735.052	−0.037	−1105.365	−0.019
3	8391.804	−0.017	4098.552	−0.012	1101.177	0.055
4	1,1058.373	−0.022	2272.798	0.016	−1135.106	−0.075
5	7492.005	−0.004	−2488.423	−0.040	−1192.864	−0.030
6	6932.435	0.039	−3323.063	−0.054	−1217.144	0.061
7	1,4103.679	0.014	2023.371	−0.049	830.587	−0.098
8	1,4146.572	−0.032	2052.973	−0.058	1682.045	0.119

**Table 6:** Registration error of selected GCPs in GCS.

No	1	2	3	4	5	6	MPE/mm	ME/mm
Best-fit method	0.063	0.057	0.070	0.071	0.080	0.108	0.185	0.078
Single constraint method	0.036	0.028	0.035	0.032	0.039	0.045	0.082	0.036
Proposed method	0.032	0.026	0.032	0.029	0.034	0.041	0.067	0.033

**Table 7:** Measuring results of sphere spacing.

No.	D1/mm		D2/mm	
	Measured Value	Sphere Spacing Error	Measured Value	Sphere Spacing Error
1	851.583	0.107	1007.531	0.113
2	851.588	0.112	1007.535	0.117
3	851.577	0.101	1007.516	0.098
4	851.586	0.110	1007.533	0.115
5	851.589	0.113	1007.534	0.116
MPE	851.589	0.113	1007.535	0.117
ME	851.584	0.108	1007.530	0.112

**Table 8:** Measurement value of feature holes centered on LMAs in GCS.

No	KLFs	Three-Dimensional Coordinates/mm
LMA 1	Hole 1 Hole 2 Hole 3 Hole 4	(7392.554, −433.481, 992.677) (7485.963, −487.790, 1058.371) (7546.156, −525.868, 910.609) (7455.501, −471.924, 846.932)
LMA 2-1	Hole 1 Hole 2 Hole 3 Hole 4	(6337.459, −359.366, 332.194) (6387.375, −367.301, 285.976) (6339.471, −335.406, 237.449) (6297.899, −332.021, 289.563)
LMA 2-2	Hole 1 Hole 2 Hole 3 Hole 4	(5383.108, 121.363, 358.896) (5436.644, 113.361, 313.480) (389.528, 141.866, 272.803) (5342.718, 146.307, 321.817)
LMA 2-3	Hole 1 Hole 2 Hole 3 Hole 4	(4386.555, 566.960, 378.743) (4426.865, 558.537, 331.288) (4392.510, 590.790, 280.258) (4345.942, 602.556, 324.067)

## Data Availability

The data presented in this study are available on request from the corresponding author.
